# Antecedents of social media addiction in high and low relational mobility societies: Motivation to expand social network and fear of reputational damage

**DOI:** 10.1371/journal.pone.0300681

**Published:** 2024-04-18

**Authors:** Shuma Iwatani, Eiichiro Watamura

**Affiliations:** 1 Graduate School of Humanities and Sociology, The University of Tokyo, Tokyo, Japan; 2 Graduate School of Human Sciences, Osaka University, Osaka, Japan; University of Rome La Sapienza: Universita degli Studi di Roma La Sapienza, ITALY

## Abstract

Contrary to previous studies on the antecedent factors of social media addiction, we focused on the social environmental factor of relational mobility (i.e., the ease of constructing new interpersonal relationships) and investigated its relationship with social media addiction. People in low relational mobility societies have fewer opportunities to select new relationship partners and consequently feel a stronger need to maintain their reputation. We hypothesized that (1) people in low relational mobility societies are more strongly addicted to social media because they estimate that greater reputational damage will be caused by ignoring messages and (2) people in low relational mobility societies estimate greater reputational damage than actual damage. We conducted two online experiments with 715 and 1,826 participants. Our results demonstrated that (1) there is no relationship between relational mobility and social media addiction and (2) people in both high and low relational mobility societies overestimate reputational damage. Furthermore, we demonstrated that the social media addiction mechanism differs between societies: (3) people in low relational mobility societies estimate greater reputational damage, whereas (4) people in high relational mobility societies are more motivated to expand their social networks; both mechanisms strengthen their social media addiction. Based on these results, we propose interventions for moderating social media addiction in both high and low relational mobility societies.

## 1. Introduction

Social media platforms have both positive and negative effects on people [1]. On the positive side, it allows people to obtain information, send messages from anywhere, and communicate more closely with others. On the negative side, some users are *addicted* to social media and spend excessive time on it. In this study, we attempted to demonstrate the mechanism through which people become addicted to social media.

The term "addiction" has various meanings, and its definition has expanded over the years. It can be classified into two main categories: substance addiction and non-substance addiction [2] (non-substance addiction is also referred to as behavioral addiction [3]). Substance addiction is a neuropsychiatric disorder characterized by a recurring desire to ingest substances, such as drugs or alcohol, despite harmful consequences [2, 4]. People who require daily intake of alcohol are defined as being addicted to alcohol. By contrast, non-substance addiction refers to addiction to things other than substances [5–7], such as pathological gambling, the Internet, and mobile phones [2].

Social media addiction is a type of non-substance addiction. The following are some definitions of social media addiction: “irrational and excessive use of social media to the extent that it interferes with other aspects of daily life” [5], “excessive use and habitual monitoring of social media, manifested in compulsive usage that comes at the expense of other activities” [8, p.747], and “being overly concerned about SNSs, to be driven by a strong motivation to log on to or use SNSs, and to devote so much time and effort to SNSs that it impairs other social activities, studies/job, interpersonal relationships, and/or psychological health and well-being” ([9], p.4054; SNS: social network service). The common items in these definitions are (i) devoting excessive time to social media and (ii) the negative consequences of using social media (i.e., interfering with other social activities such as studying, job, interpersonal relationships, psychological health, and well-being).

Social media addiction has various negative effects, including damaging mental health [5], poor life satisfaction [10], and chronic physical issues, such as neck pain or headaches [11]. Some studies that focused on company employees have demonstrated that social media addiction leads to a reduction in sleeping hours [12], increased distraction at workplace [12], and impaired productivity [8].

In this study, we used the term “addiction” or “social media addiction” in a non-clinical sense. This is because social media addiction is not included in the DSM-5-TR classification [13] and no study has demonstrated that social media addiction can have severe physical consequences [14].

### 1.1. Antecedents of social media addiction

Previous studies have investigated various antecedents of social media addiction such as neuroticism [15], lack of self-control [16], and extraversion [17], and have several perspectives on social media addiction [18]. One perspective focuses on dispositional differences such as attachment styles. D’Arienzo et al. [19] concluded that avoidant or insecure attachment style is associated with stronger social media addiction. Additionally, an empirical study by Ballarotto et al. [20] demonstrated that individuals who are less attached to their parents are more strongly addicted to Instagram. Eroglu [21] showed that people with insecure attachments (i.e., those having negative “internally working models about both themselves and others” [21] p.151) are more strongly addicted to Facebook. Additionally, Monacis et al. [22] demonstrated that people with avoidant attachment style (i.e., those who experience discomfort with intimacy) are more strongly addicted to social media.

Furthermore, some studies have focused on the motivation to use social media. For example, those who feel lonely are more strongly addicted to social media [23] as they are motivated to connect with others [24]. Moreover, extraverts are more strongly addicted to social media [17] as they use it to expand their social connections [25]. Additionally, those with a higher motivation to expand their social network would be more strongly addicted to social media, as it allows them to maintain or expand their social network.

Additionally, demographic variables, such as sex and age, may be related to social media addiction Mari et al. [26] found that females are more strongly addicted to the Internet, whereas Su et al. [27] and Alnjadat et al. [28] found that males are more strongly addicted to the Internet or social media. Moreover, age is related to social media addiction as younger individuals are more strongly addicted to social media [25].

Also, distressing changes in social situations, such as those during and after the COVID-19 pandemic, may also strengthen social media addiction. Recent studies have noted that the importance of social media as a medium for rapid information dissemination has increased after COVID-19 [29] and demonstrated that psychological distress owing to COVID-19 has strengthened social media [30], Internet [20], and Instagram [20] addictions, and social media addiction has also increased the likelihood of experiencing depression [31]. These studies imply that distressing situations and social media addiction have mutually strengthened each other, especially after the COVID-19 pandemic.

Although these studies focused on micro-level factors, such as depression or distress, the effect of macro-level social environmental factors on social media addiction is understudied and must be further investigated [18]. Based on Sun et al. [18]’s suggestion, we focused on a social environmental factor (i.e., relational mobility [32]) and investigated the relationship between the social environment and social media addiction.

We conducted two studies to examine the effect of the social environment (i.e., relational mobility) on social media addiction. Relational mobility of a society refers to how easily people in the society can select new relationship partners when necessary [32]. Relational mobility is lower in typical rural areas wherein interpersonal relationships are closed to outsiders. As relational mobility affects the sensitivity of an individual to social rejection [33, 34], it can affect their interpersonal behavior. As an example of interpersonal behavior on social media, we focused on a message exchange situation and examined whether the estimation of reputational damage incurred by ignoring messages moderates the relationship between relational mobility and social media addiction.

**Fig 1:**
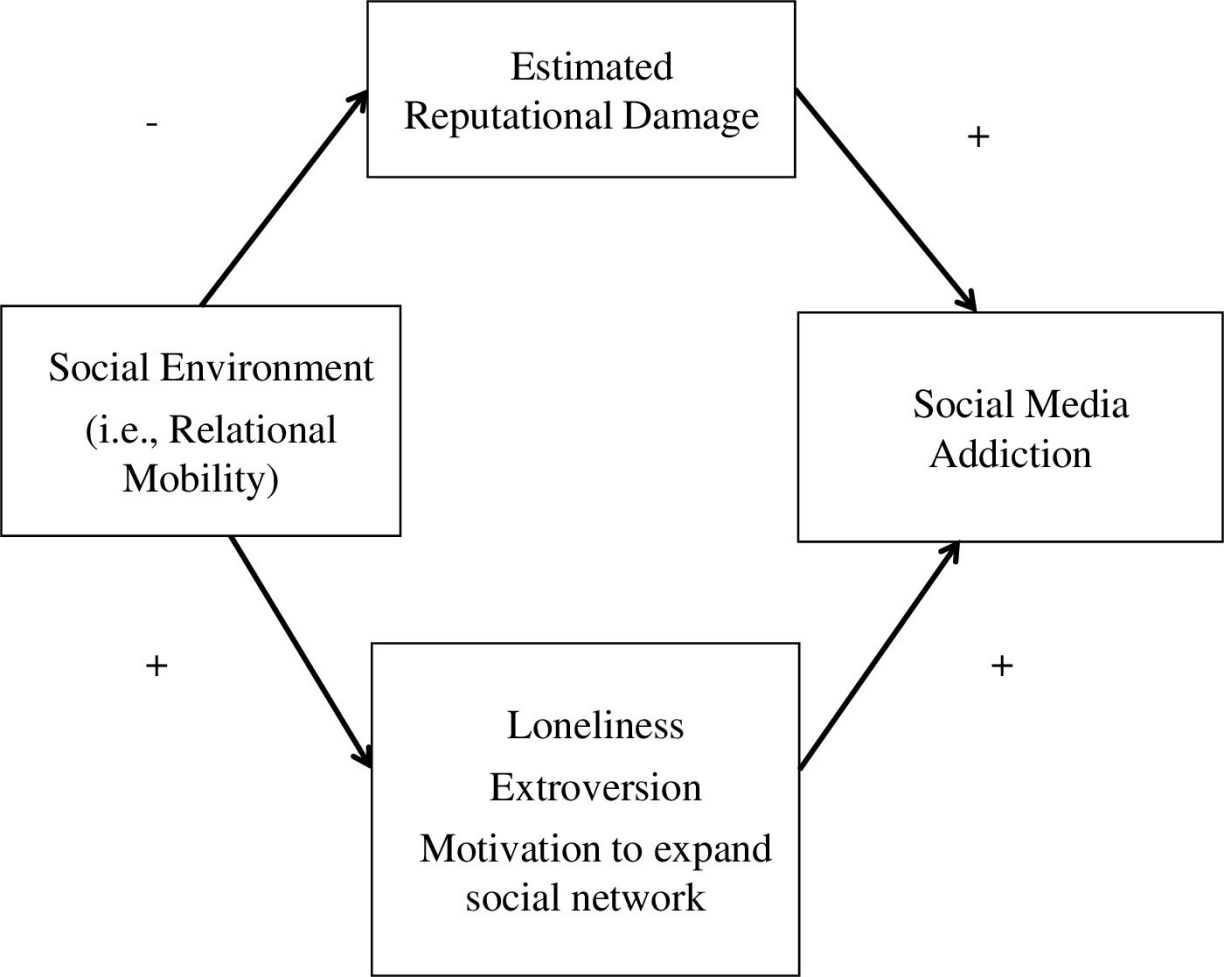
Conceptual model.

Fig 1 illustrates our conceptual model. In Study 1, we examined the following mediation process: people in lower relational mobility societies estimate that greater reputational damage is incurred by message ignorance, which strengthens their social media addiction. This model was proposed based on previous studies that have indicated that people in lower relational mobility societies are more sensitive to social rejection [33, 34], and they make decisions based on their estimations of others’ attitudes [35].

In Study 2, we additionally examined the following dual paths: (1) the mediating effect of estimated reputational damage on the *negative* relationship between relational mobility and social media addiction and (2) the mediating effect of extroversion, the motivation to expand the social network, and loneliness on the *positive* relationship between relational mobility and social media addiction. We focused on extroversion, motivation to expand social network, and loneliness because they are related to both relational mobility and social media addiction.

In the following section, we review studies on relational mobility, develop our hypotheses, and outline our contributions.

### 1.2. Relational mobility and social media addiction

We first focused on the social environmental factor of *relational mobility* [32], which is a sociological variable that refers to “the amount of opportunities people have in a given society or social context to select new relationship partners when necessary” [32]. Relational mobility is lower in typical rural areas, where people exclusively develop intimate relationships with neighbors and seldom construct new relationships with outsiders, whereas it is higher in typical urban areas, where people have weaker social ties and entering or leaving relationships is easier.

Thus, people in low relational mobility societies cannot easily construct alternate relationships, even when they earn a bad reputation and are excluded from their communities. Therefore, the consequences of earning a bad reputation are worse for people in low relational mobility societies [36], which strengthens their need to avoid reputational damage. Indeed, people in lower relational mobility societies are more sensitive to social rejection [33, 34] and refrain from sharing personal information, such as embarrassing experiences or failures [37].

Based on these studies, we assume that people in lower relational mobility societies are more strongly addicted to social media. Additionally, as they are more sensitive to social rejection, they have more difficulty ignoring messages on social media and thus spend more time on social media, resulting in higher addiction.

*Hypothesis 1*: People in low relational mobility societies are more strongly addicted to social media.

### 1.3. Mediating effect of estimated reputational damage

People in lower relational mobility societies estimate greater reputational damage incurred by ignoring messages on social media because, as described in the Section 1.2., they are more sensitive to social rejection [33, 34]. In some cases, this sensitivity might result in an overestimation of reputational damage, leading them to unnecessarily respond to messages on social media. However, in other cases, this sensitivity could reduce the possibility that they underestimate the damage in situations where ignoring them could lower their reputation, and mistakenly ignore messages and damage their reputation. Therefore, estimating greater reputational damage and refraining from ignoring messages are adaptive in that this estimation (*overestimation* in some cases) can lower the possibility of damaging their reputation. However, this estimation can strengthen their addiction to social media. Because forming and maintaining strong and stable interpersonal relationships is important to humans [38], people make decisions based on their estimations of others’ attitudes [35]. For example, people are more likely to follow norms when they estimate that deviating from them will tarnish their reputation [39]. Based on these studies, we assumed that people in lower relational mobility societies estimate greater reputational damage incurred by ignoring messages, which strengthens their social media addiction.

*Hypothesis 2*: The estimation of greater reputational damage incurred by ignoring messages mediates the negative relationship between relational mobility and social media addiction.

### 1.4. Accuracy of reputational damage estimation

Hypothesis 2 focuses on the mediating effect of estimated reputational damage. In this section, we examine two questions: do people in both high and low relational mobility societies accurately estimate reputational damage?

Based on [36], we hypothesized that people in low relational mobility societies overestimate the possibility of earning a bad reputation. Overestimation of reputational damage can help them avoid situations wherein they mistakenly estimate that performing detrimental actions would *not* damage their reputation when it would. An example of this situation on social media is that people mistakenly estimate that ignoring messages will *not* tarnish their reputation, even if it does. If they ignore messages based on this underestimation, they will gain a bad reputation, the cost of which is higher in lower relational mobility societies. Therefore, people in lower relational mobility societies are more likely to overestimate reputational damage, especially because interpersonal relationships in these societies are closed and the cost of earning a bad reputation is higher. Indeed, Iwatani and Muramoto [39] focused on community activities, such as cleanup drives, and demonstrated that people in low relational mobility societies overestimate the possibility of gaining a bad reputation, whereas those in high relational mobility societies estimate it accurately. In this study, we extend their findings to the context of social media and investigate the following hypothesis:

*Hypothesis 3*: People in low relational mobility societies overestimate the possibility of receiving a bad evaluation by ignoring messages, whereas people in high relational mobility societies do not.

### 1.5. Contributions of this study

We believe our study has at least three original contributions. First, it highlights the effects of the social environment on social media addiction. The human mind, including cognition, emotion, and motivation, is affected by both cultural [40] and social environments [41]; therefore, social environmental factors can impact social media addiction. However, few studies have investigated the effect of social environments on social media addiction [42] (however, see [43], wherein the effect of relational mobility on problematic Internet use was investigated). The novelty of this study lies in its investigation of social media addiction from the perspective of socio-ecological psychology.

Second, we focus on interpersonal interactions between social media users as an antecedent of social media addiction, whereas previous studies have primarily focused on individual psychological factors [15–17]. The originality of our study lies in the fact that we focus on miscommunications between social media users, that is, inaccurately (over)estimated reputational damage as an antecedent factor of social media addiction.

Third, our study also features originality for studies on socio-ecological psychology in that we investigate the effect of relational mobility on online behavior. Previous studies have demonstrated the effect of relationality on general trust, self-esteem, and intimacy with close friends [44]. However, few studies have demonstrated the effect of offline relational mobility on the online psychological tendencies of humans, except for Dong et al. [43] and Thomson et al. [45], who examined the effect of relational mobility on problematic Internet use or online privacy concerns of people.

We conducted two studies to examine the following hypotheses: (1) the direct effect of the social environment (relational mobility) on social media addiction, (2) the mediating effect of reputational damage estimation on the relationship between the social environment and social media addiction, and (3) the accuracy of reputational damage estimation.

## 2. Materials and methods

### 2.1. Study 1

We tested the proposed hypotheses using the LINE message exchange service, which is the most popular social media platform in Japan, wherein it is used by approximately 80% of the Internet users [46]. LINE was considered suitable for this study because it provides a "read notification function," through which users can determine whether their messages have been read and ignored. Additionally, they are aware that their messaging partner can determine if their messages are ignored.

The experiment included two conditions: (1) wherein participants ignored messages (ignorer condition) and (2) wherein participants’ messages were ignored (ignored condition). In the ignorer condition, participants imagined a scenario wherein they read the messages received on LINE and ignored them. They estimated how message senders would evaluate the participants when participants themselves ignored messages (estimated reputational damage). In the ignored condition, participants imagined a scenario wherein they sent messages and the receiver read and ignored them, and evaluated the receiver who ignored the messages (actual reputational damage). We compared the estimated and actual reputational damage and investigated whether people from lower relational mobility societies estimated more reputational damage than the actual damage. We also investigated whether people from lower relational mobility societies estimated higher reputational damage and were more strongly addicted to social media.

#### 2.1.1. Participants

This study was approved by the Ethics Review Committee of the University of Tokyo. Written informed consent was obtained from all participants. They were recruited through a crowdsourcing service (Yahoo! Crowdsourcing; https://crowdsourcing.yahoo.co.jp/) between February 19 and 20, 2022. They were informed of the purpose of this study, and only those who agreed to participate (i.e., those who clicked “agree”) proceeded to answer the questions. Study 1 included 715 participants.

Study 1 was conducted using the between-participant design. We excluded 54 participants who did not pass the instructional manipulation check [47], 126 who did not use LINE, and 27 who had no friends whom they could contact privately through LINE. We also excluded data with missing values and one participant who answered that their age was 3. Finally, we analyzed the data of 453 participants (males: 308, females: 138, and others: 7). Their average age was 46.46 years (*SD* = 11.17).

Half of the participants were randomly assigned to the *ignorer* condition, and the other half were randomly assigned to the *ignored* condition, resulting in 222 and 231 participants assigned to the *ignorer* and *ignored* conditions, respectively.

We examined whether the sample size was sufficiently large using G*Power version 3.1.9.7 [48] to conduct a post-hoc power analysis, assuming *f* = 0.05 (small to medium effect size), α = 0.05, *N* = 453, and three predictors (condition, relational mobility, and the interaction between them). The calculated power of the test was 0.99, which indicated that the sample size was adequate.

#### 2.1.2. Reputational damage estimation (ignorer condition)

The participants were first asked to write the first-name initials of one of their friends they had privately contacted. The friend’s name is denoted as Mr. A in this study (It was denoted as “A-san” in our actual question). Participants assigned to the ignorer condition were asked to read and imagine a scenario wherein they received a message from Mr. A that stated that they had to discuss something, read it, but did not reply for two or three days.

After participants read the scenario, they estimated Mr. A’s evaluation of them by answering the following six items, extracted from a previous study [49], on a six-point Likert scale, ranging from 1 (“strongly disagree”) to 6 (“strongly agree”): “Mr. A would think you are a bad person,” “Mr. A would think you are an untrustworthy person,” “Mr. A would think you are an honest person,” “Mr. A would think they do not want to be your friend anymore,” “Mr. A would think they cannot feel secure with you,” and “Mr. A would think you are a cunning person.” We calculated the reputational damage estimation score by averaging the sum of the scores (α = 0.89, *M* = 2.89, *SD* = 0.99).

#### 2.1.3. Participants’ evaluation (ignored condition)

Participants assigned to the ignored condition were asked to read and imagine a scenario wherein they sent a message to Mr. A stating that they had to discuss something, Mr. A received and read it but did not reply for two or three days.

After reading the scenario, participants answered questions regarding their evaluation of Mr. A. The items were almost the same as those in the previous scenario, and only the subjects were changed. For example, we changed the item “Mr. A would think you are a bad person” to “I think Mr. A is a bad person.” We again calculated the evaluation score by averaging the sum of the scores (α = 0.89, *M* = 2.26, *SD* = 0.88).

#### 2.1.4. Social media addiction

We used the social media addiction questionnaire (SMAQ; 7-point scale), which is composed of eight items and was proposed by Hawi and Samaha [10]. We changed the term “social media” in SMAQ to “LINE” for this study. For example, the question “I often think about *social media* when I am not using it” was modified to “I often think about *LINE* when I am not using it.” As in [10], we calculated the social media addiction score by averaging the sum of the scores (α = 0.86, *M* = 2.73, *SD* = 1.02). As there was no threshold to distinguish between those addicted to social media and those who were not [10], we did not perform threshold-based distinguishing between those who were addicted to LINE and those who were not. Participants were considered to be more strongly addicted to LINE if they scored higher on this scale.

#### 2.1.5. Relational mobility

Relational mobility was measured using the relational mobility scale [32]. Participants were presented with 12 statements and asked how much they agreed with them based on a six-point Likert scale, from 1 (“strongly disagree”) to 6 (“strongly agree”). The statements included the following: “they (people in the immediate society (your school, workplace, town, neighborhood, etc.) in which you live) have many chances to get to know other people.” The relational mobility score was calculated by averaging the sums of the scores (α = 0.75, *M* = 3.60, *SD* = 0.51). The relational mobility of the participant’s society was considered to be higher if they scored higher on this scale.

### 2.2. Study 2

Although Study 1 only investigated the factors that mediate the *negative* relationship between relational mobility and social media addiction, Study 2 investigated the factors that mediate the *positive* relationship between the two. We focused on the following three factors: loneliness, extroversion, and the motivation to expand social network. We examined whether these three factors mediated the *positive* relationship between relational mobility and social media addiction, which would cancel out the *negative* relationship examined in Study 1.

First, we focused on loneliness. We assumed that the relationship between relational mobility and loneliness was positive based on the study by Oishi et al. [50], which demonstrated that people in mobile conditions (wherein they imagined that they would move to a different location every other year) experienced more loneliness than those in stable conditions (wherein they imagined that they would stay in the same city for at least ten years). Additionally, there is a positive relationship between loneliness and social media addiction [23], which suggests that loneliness mediates a positive relationship between relational mobility and social media addiction.

Next, we focused on extroversion. There is a positive relationship between the within-state migration level and extroversion [51], which implies that there is a positive relationship between relational mobility and extroversion. Additionally, there is a positive relationship between extroversion and social media addiction [17]. These findings suggest that extroversion mediates the positive relationship between relational mobility and social media addiction.

Finally, we focus on the motivation to expand social network. An experimental study demonstrated that people in the mobile condition are more motivated to expand their social networks than those in the stable condition [50]. In addition, extraverts have larger social networks, which can promote their use of social media [4]. These findings suggest that the motivation to expand the social network mediates the positive relationship between relational mobility and social media addiction. In summary, we developed the following additional hypotheses and examined the model presented in Fig 1.

*Hypothesis 4a*: Loneliness mediates the positive relationship between relational mobility and social media addiction.
*Hypothesis 4b*: Extroversion mediates the positive relationship between relational mobility and social media addiction.
*Hypothesis 4c*: Motivation to expand social network mediates the positive relationship between relational mobility and social media addiction.

#### 2.2.1. Participants

This study was approved by the Ethics Review Committee of the University of Tokyo. Written informed consent was obtained from all the participants. Study 2 employed the within-participants design and included 1826 participants, recruited through the same crowdsourcing service (Yahoo! Crowdsourcing; https://crowdsourcing.yahoo.co.jp/) between August 26 and 27, 2022. They were informed of the purpose of this study, and only those who agreed to participate proceeded to answer the questions. We excluded 143 participants who did not pass the instructional manipulation check [47], 309 who did not use LINE, and 209 who had no friends whom they could contact privately through LINE. We also excluded data with missing values and eventually analyzed the data of 1065 participants (males: 670, females: 374, and others: 21). Their average age was 48.11 years (*SD* = 12.09).

We examined whether the sample size was sufficiently large by conducting a post-hoc power analysis, assuming a root mean square error of approximation (RMSEA) in the null hypothesis = 0.05, RMSEA in the alternative hypothesis = 0.01, degrees of freedom = 7, *N* = 1065, and α = 0.05. The calculated power was 0.85, which indicated that the sample size was adequate.

#### 2.2.2. Measurements

As in Study 1, participants were asked to write the first-name initials of their friends they had privately contacted, who were denoted as Mr. A. Thereafter, they were asked to imagine the following scenarios: (1) wherein they ignored messages and (2) wherein their messages were ignored.

#### 2.2.3. Reputation damage estimation

The participants read the same scenario as in Study 1 (ignorer condition), wherein they ignored Mr. A’s message, and answered the following five items on a six-point Likert scale based on a previous study [49], ranging from 1 (“strongly disagree”) to 6 (“strongly agree”): “Mr. A would think you are a bad person,” “Mr. A would think you are an untrustworthy person,” “Mr. A would think they cannot feel secure with you,” “Mr. A would think you are an unreliable person,” and “Mr. A would not want to deepen their friendship with you.” We calculated the reputational damage estimation score by averaging the sums of the scores (α = 0.96, *M* = 3.16, *SD* = 1.19).

#### 2.2.4. Participants’ evaluation

Next, the participants read the same scenario as in Study 1 (ignored condition), wherein Mr. A ignored their messages. Thereafter, they responded with their evaluations of Mr. A. These items were almost the same as those mentioned above, and only their subjects were changed. For example, we changed the item “Mr. A would think you are a bad person” to “I think Mr. A is a bad person.” We calculated the evaluation score by averaging the sum of the scores (α = 0.96, *M* = 2.68, *SD* = 1.14).

#### 2.2.5. Social media addiction

We used the same questionnaires as in Study 1 to calculate the social media addiction scores. The sum of the scores were averaged (α = 0.86, *M* = 2.69, *SD* = 1.03).

#### 2.2.6. Extroversion

We measured extroversion using the Ten-Item Personality Inventory assessment [52]. The participants were asked to answer the following two items on a seven-point Likert scale, ranging from 1 (“strongly disagree”) to 7 (“strongly agree”): “I see myself as extraverted, enthusiastic” and “I see myself as reserved, quiet” (reverse-scored item). We calculated the extroversion score by averaging the sums of the scores (*r* = 0.47, *p* < 0.01, *M* = 3.55, *SD* = 1.31). A participant was considered more extraverted if they scored higher.

#### 2.2.7. Motivation to expand social network

We measured the motivation to expand the social network using a seven-point Likert scale [50]. The questionnaire was composed of four items (e.g., “eager to make friends,” “want to meet new people”). We calculated the motivation to expand the social network by averaging the sum of the scores (α = 0.92, *M* = 3.52, *SD* = 1.37). Participants were considered to have higher motivation to expand their social networks if they scored higher on this scale.

#### 2.2.8. Loneliness

We measured loneliness using a five-point Likert scale [53]. The scale was composed of six statements (e.g., “I usually sense an experience of emptiness,” “I often feel missing close people around me”). We calculated the loneliness score by averaging the sum of the scores (α = 0.85, *M* = 2.82, *SD* = 0.76). Participants were considered to be lonelier if they scored higher on this scale.

#### 2.2.9. Relational mobility

As stated in Section 2.1.5, relational mobility score was measured using the relational mobility scale [32]. It was calculated by averaging the sum of the scores (α = 0.78, *M* = 3.66, *SD* = 0.53).

### 2.3. Statistical analysis

We used R version 4.3.2 for statistical analyses. For mediation analyses, multiple regression analysis, and generalized linear mixed model analysis, the statistical significance standard was set as *p* = .05, whereas for structural equation modeling (SEM), the statistical significance standard for the model fit was set as RMSEA = .05.

Study 1 was conducted using a between-participants design (*ignorer* and *ignored* conditions). To test Hypotheses 1 and 2, we analyzed participants in the *ignorer* condition, (i.e., those who answered reputational damage estimation) and conducted a mediation analysis using the bootstrap method (5000 samples) to examine whether the negative effect of relational mobility on social media addiction was mediated by reputational damage estimation. This analysis was conducted after centering all variables in the model.

For testing Hypothesis 3, we conducted a multiple regression analysis. The evaluation was predicted using a dummy evaluator variable (*ignored* condition (i.e., participants’ actual reputational damage) = 0, *ignorer* condition (i.e., estimated reputational damage from others) = 1), relational mobility, and the interaction between them. This analysis was also conducted after centering all variables in this model.

Study 2 employed a within-participant design. Participants read both the *ignorer* and *ignored* condition scenarios. For testing Hypothesis 2 and Hypotheses 4a–c, we employed SEM techniques and examined the following hypotheses: estimation of greater reputational damage mediates the negative relationship between relational mobility and social media addiction, whereas loneliness, extroversion, and motivation to expand social networks mediates the positive relationship between them (Fig 1).

For testing Hypothesis 3, we used a generalized linear mixed model with random intercepts for the participants to examine our hypothetical model. The evaluation toward the *ignorer* was predicted using the dummy evaluator variable (*ignored* condition = 0, *ignorer* condition = 1), relational mobility, and the interaction between them. This analysis was conducted after centering all variables in this model.

## 3. Results and discussion

### 3.1. Study 1

#### 3.1.1. Are people in lower relational mobility societies more addicted to social media?

First, we examined Hypotheses 1 and 2: (1) people in low relational mobility societies are more strongly addicted to social media (Hypothesis 1) and (2) the estimation of greater reputational damage incurred by ignoring messages would mediate the negative relationship between relational mobility and social media addiction (Hypothesis 2).

We only analyzed the answers of participants in the *ignorer* condition because we did not measure the estimated reputational damage in the *ignored* condition. After centering all variables in the model, we conducted a mediation analysis using the bootstrap method (5000 samples) to examine whether the effect of relational mobility on social media addiction was mediated by reputational damage estimation. Relational mobility had a significant effect on reputational damage estimation, indicating that people in low relational mobility societies estimated a higher reputational damage caused by ignoring messages (β = -0.15, *p* = 0.04). The effect of reputational damage estimation on social media addiction was not statistically significant (β = 0.14, *p* = 0.06). Additionally, the direct effect of relational mobility on social media addiction was not significant (β = -0.10, *p* = 0.18). These results did not support Hypotheses 1 and 2. However, consistent with Hypothesis 2, there was a statistically significant negative correlation between relational mobility and reputational damage estimation (*r* = -0.15, *p* = 0.03) as well as a statistically significant positive correlation between reputational damage estimation and social media addiction (*r* = 0.16, *p* = 0.02), although there was no statistically significant correlation between relational mobility and social media addiction (*r* = -0.12, *p* = 0.07).

Thereafter, we conducted an additional analysis using age and sex (male = 0, female = 1) as control variables. To examine the effect of sex, seven participants who did not answer “male” or “female” were excluded. The effect of relational mobility on reputational damage estimation was statistically significant (β = -0.14, *p* = 0.05). In contrast to the aforementioned analysis, the effect of reputational damage estimation on social media addiction was also statistically significant (β = 0.15, *p* = 0.04). The direct effect of relational mobility on social media addiction was not significant (β = -0.09, *p* = 0.28) as in the above analysis. Additionally, the main effect of sex (β = 0.14, *p* = 0.03) was statistically significant, whereas that of age (β = -0.09, *p* = 0.23) was not.

#### 3.1.2. Do people in low relational mobility societies overestimate reputational damage?

Next, we examined Hypothesis 3: people in low relational mobility societies overestimate the possibility of receiving a bad evaluation incurred by ignoring messages, whereas people in high relational mobility societies do not.

Prior to the analysis, we constructed a dummy variable for the *evaluator* (participants’ actual reputational damage = 0, estimated reputational damage from others = 1). After centering all variables in this model, we conducted a multiple regression analysis. The evaluation was predicted using the dummy *evaluator* variable, relational mobility, and the interaction between the two.

The main effect of *evaluator* was significant (β = 0.31, *p* < 0.01), but the interaction effect between the evaluator and relational mobility was not (β = -0.02, *p* = 0.60). These results imply that people in low relational mobility societies estimate greater reputational damage incurred by ignoring messages than the actual damage (consistent with Hypothesis 3), and the same holds true for people in high relational mobility societies (inconsistent with Hypothesis 3).

The main effect of relational mobility was also significant (β = -0.13, *p* < 0.01), which implies that (1) people who ignored messages were evaluated more negatively in low relational mobility societies than in high relational mobility societies and (2) people in low relational mobility societies estimated that ignoring messages would incur higher reputational damage than people in high relational mobility societies.

We conducted an additional analysis using age and sex (male = 0, female = 1) as control variables. To examine the effect of sex, seven participants who did not answer “male” or “female” were excluded. The results were the same as those obtained in the aforementioned analysis. The main effects of the *evaluator* (β = 0.31, *p* < 0.01) and relational mobility (β = -0.12, *p* = 0.01) were statistically significant, whereas the interaction effect between the *evaluator* and relational mobility was not (β = -0.03, *p* = 0.56). Additionally, the main effects of age (β = -0.08, *p* = 0.07) and sex (β = -0.02, *p* = 0.68) were not significant.

#### 3.1.3. Moderating effect of age

We exploratively examined the moderating effect of age; whether the effect of reputational damage estimation on social media addiction differed depending on age. A multiple regression analysis was conducted after entering all variables in the model. Social media addiction was used as the dependent variable, whereas relational mobility, reputational damage estimation, sex, age, and the interaction between reputational damage estimation and age were used as independent variables. The main effects of reputational damage estimation (β = 0.15, *p* = 0.02) and sex (β = 0.14, *p* = 0.04) were statistically significant, whereas those of relational mobility (β = -0.08, *p* = 0.22) and age (β = -0.09, *p* = 0.20) were not. Additionally, the interaction effect was not significant (β = 0.01, *p* = 0.84).

#### 3.1.4. Discussion

In Study 1, we examined (1) the effect of relational mobility on social media addiction (Hypothesis 1), (2) mediating effect of reputational damage estimation on the relationship between relational mobility and addiction (Hypothesis 2), and (3) accuracy of reputational damage estimation (Hypothesis 3).

First, we found that both relational mobility and reputational damage estimation had no effect on social media addiction; these results do not support Hypothesis 1. Second, we found that people in lower relational mobility societies estimated higher reputational damage. We also found a positive correlation between reputational damage estimation and social media addiction. These results were consistent with Hypothesis 2 (the mediating effect of reputational damage estimation), but this hypothesis was not supported because no direct relationship between relational mobility and social media addiction was observed. These results imply that other factors mediate the *positive* relationship between relational mobility and social media addiction, which negates the negative relationship between them. We further examined this possibility in Study 2. Third, we found that people overestimate the reputational damage caused by ignoring messages. This result partially supported Hypothesis 3, in that people in low relational mobility societies overestimate reputational damage incurred by ignoring messages, but contradicted Hypothesis 3, in that people in high relational mobility societies also overestimate it.

### 3.2. Study 2

#### 3.2.1. Relationship between relational mobility and social media addiction

We investigated the relationship between relational mobility and social media addiction using SEM techniques and examined the following possibilities: estimation of greater reputational damage mediates a negative relationship between relational mobility and social media addiction (Hypothesis 2), whereas (2) loneliness, extroversion, and motivation to expand social networks mediate a positive relationship between relational mobility and social media addiction (Hypotheses 4a, 4b, and 4c; Fig 1). However, the model did not fit the data (RMSEA = 0.24).

In this model, we focused on three factors that would mediate the positive relationship between relational mobility and social media addiction (Hypotheses 4a, 4b, and 4c): loneliness, extroversion, and the motivation to expand the social network. Indeed, extroversion and the motivation to expand the social network were significantly and positively correlated with social media addiction (*r* = 0.12, *p* < 0.01; *r* = 0.28, *p* < 0.01), but loneliness was not (*r* = 0.04, *p* = 0.23).

**Fig 2:**
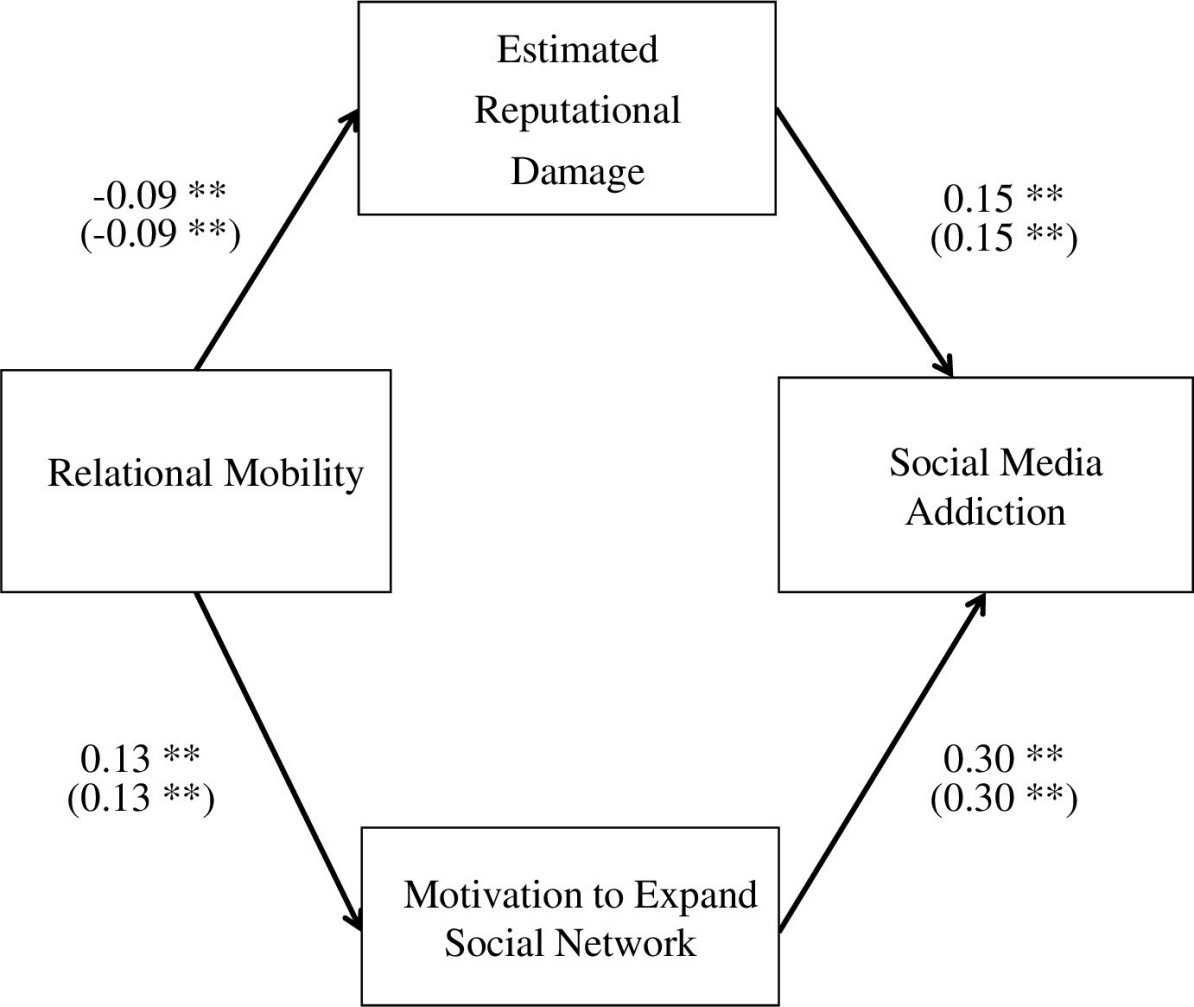
Two pathways to social media addiction (SEM results). Note**: *p* < 0.01; the values in parentheses indicate the results of the additional analyses with covariates (i.e., age and gender).

Therefore, we focused only on the motivation to expand the social network, as it had the strongest correlation with social media addiction. We used SEM techniques and examined the following possibilities: (1) estimation of greater reputational damage incurred by ignoring messages mediates the negative relationship between relational mobility and social media addiction (Hypothesis 2) and (2) motivation to expand social network mediates the positive relationship between them (Hypothesis 4c). This model fit the data (RMSEA = 0.05; Fig 2). We conducted an additional analysis using age and sex (male = 0, female = 1) as control variables. To examine the effect of sex, we excluded 21 participants who did not answer “male” or “female.” The result was the same as that of the aforementioned analysis (RMSEA = 0.05; Fig 2). The effect of sex on social media addiction was statistically significant (β = 0.08, *p* = 0.01), whereas that of age was not (β = 0.01, *p* = 0.85).

**Fig 3:**
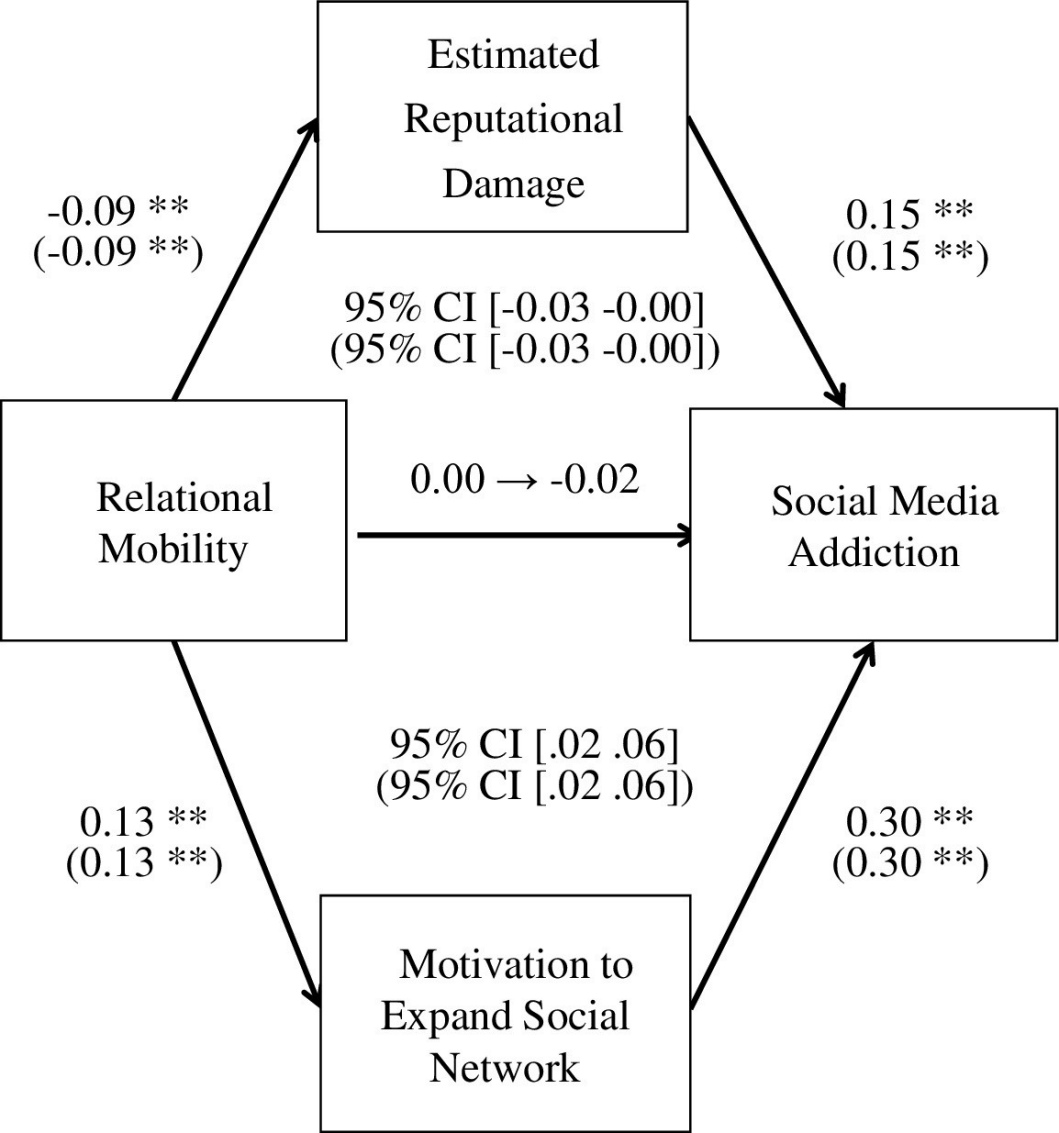
Mediating effect of reputational damage estimation and the motivation to expand the social network on the relationship between relational mobility and social media addiction (dual mediation analysis). Note**: *p* < 0.01; the values in parentheses indicate the results of the additional analyses with covariates (i.e., age and gender).

Based on these results, we conducted a dual mediation analysis using 1000 bootstrap samples. The results indicated that reputational damage estimation mediated the negative relationship between relational mobility and social media addiction (indirect effect = -0.01, *p* = 0.03), whereas the motivation to expand social network mediated the positive relationship (indirect effect = 0.04, *p* < 0.01; Fig 3). We also conducted an additional analysis using age and sex (male = 0, female = 1) as control variables, and obtained same result as that in the aforementioned analysis; reputational damage estimation mediated the negative relationship between relational mobility and social media addiction (indirect effect = -0.01, *p* = 0.03), whereas the motivation to expand social network mediated the positive relationship (indirect effect = 0.04, *p* < 0.01; Fig 3). These results support Hypotheses 2 and 4c.

When we used extroversion or loneliness instead of motivation to expand the social network, the models did not fit the data (RMSEA = 0.12 and 0.20, respectively). The models did not fit when entering the control variables (age and gender), either (RMSEA = 0.09 and 0.14, respectively).

#### 3.2.2. Do people in low relational mobility societies overestimate their reputational damage?

Next, we examine Hypothesis 3. We conducted a generalized linear mixed model analysis in Study 2, although we conducted a multiple regression analysis in Study 1. This is because Study 2 employed the within-participants design, whereas Study 1 employed the between-participants design.

Prior to the analysis, we constructed a dummy variable for the *evaluator* (participants’ actual evaluations = 0, evaluation from others = 1). After centering all variables in the model, we used a generalized linear mixed model with random intercepts for participants to examine our hypothetical model. The evaluation was predicted using the dummy evaluator variable, a relational mobility variable, and the interaction between the two.

The main effect of the *evaluator* was significant (β = 0.20, *p* < 0.01), but the interaction effect between the evaluator and relational mobility was not (β = 0.01, *p* = 0.49). These results imply that people in low relational mobility societies estimate that greater reputational damage will be incurred by ignoring messages than the actual damage (consistent with Hypothesis 3), and the same hold true for people in high relational mobility societies (inconsistent with Hypothesis 3).

The main effect of relational mobility was also significant (β = -0.10, *p* < 0.01), which implies that (1) people in low relational mobility societies evaluated those who ignored messages more negatively than those in high relational mobility societies, and (2) people in low relational mobility societies also estimated that ignoring messages would incur higher reputational damage than those in high relational mobility societies.

We conducted an additional analysis using age and sex (male = 0, female = 1) as control variables and obtained the same results as those in the aforementioned analysis. The main effects of the *evaluator* (β = 0.20, *p* < 0.01) and relational mobility (β = -0.10, *p* < 0.01) were statistically significant, whereas the interaction effect between them was not (β = 0.01, *p* = 0.50). Additionally, the main effects of age (β = -0.04, *p* = 0.14) and sex (β = -0.02, *p* = 0.39) were not significant.

#### 3.2.3. Moderating effect of age

We exploratively examined the moderating effect of age; whether the effect of reputational damage estimation on social media addiction and that of the motivation to expand the social network differs depending on age. We conducted a multiple regression analysis after centering all variables in the model. Social media addiction was used as the dependent variable, whereas relational mobility, reputational damage estimation, the motivation to expand the social network, sex, age, the interaction between reputational damage estimation and age, and the interaction between the motivation to expand the social network and age as independent variables. The main effects of reputational damage estimation (β = 0.15, *p* < 0.01) and the motivation to expand the social network (β = 0.30, *p* < 0.01) were statistically significant, whereas those of relational mobility (β = -0.02, *p* = 0.42), age (β = 0.00, *p* = 0.90), and sex (β = 0.05, *p* = 0.07) were not. Additionally, the interaction effect between reputational damage estimation and age was not statistically significant (β = -0.01, *p* = 0.70), whereas that between the motivation to expand the social network and age was statistically significant (β = -0.06, *p* = 0.03). The effect of the motivation to expand the social network was greater among younger participants (*M* - 1*SD*; β = 0.36, *p* < 0.01) than among older (*M* + 1*SD*; β = 0.24, *p* < 0.01).

#### 3.2.4. Discussion

Study 2 hypothesized that (1) reputational damage estimation mediates the negative relationship between relational mobility and social media addiction, whereas (2) loneliness, extroversion, and the motivation to expand the social network mediate the positive relationship. We hypothesized that these two mediations would cancel each other out; therefore, there will be no relationship between relational mobility and social media addiction.

We tested these hypotheses using SEM; however, they were not supported. We additionally tested another model that focused only on the motivation to expand the social network: (1) reputational damage estimation mediates the negative relationship between relational mobility and social media addiction and (2) motivation to expand the social network mediates the positive relationship. This model was supported, which implies that the factors promoting social media addiction differ between high and low relational mobility societies. Reputational damage estimation strengthens social media addiction in low relational mobility societies, whereas the motivation to expand social networks strengthens it in high relational mobility societies.

### 3.3. General discussion

#### 3.3.1. Summary of results

We focused on message exchanges on social media and investigated the effect of social environment (relational mobility) on social media addiction. In Study 1, we examined the following hypotheses: (1) people in low relational mobility societies are more strongly addicted to social media (Hypothesis 1), (2) the estimation of greater reputational damage incurred by ignoring messages mediates the negative relationship between relational mobility and social media addiction (Hypothesis 2), and (3) people in low relational mobility societies overestimate the possibility of receiving a bad evaluation, whereas people in high relational mobility societies do not. In Study 2, we additionally examined (4) loneliness, extroversion, and the motivation to expand the social network mediate the positive relationship between relational mobility and social media addiction (Hypotheses 4a, 4b, and 4c).

Hypotheses 1 and 2 were not supported; we conducted a mediation analysis but observed no relationship between relational mobility and social media addiction or between reputational damage estimation and social media addiction. In contrast, when we conducted the correlational analyses, although we found no statistically significant correlation between relational mobility and social media addiction, we found a statistically significant negative correlation between relational mobility and reputational damage estimation, as well as a statistically significant positive correlation between reputational damage estimation and social media addiction. These results are partially consistent with Hypothesis 2 and imply that other factors mediate the *positive* relationship between relational mobility and social media addiction, which might negate the negative relationship hypothesized in Hypothesis 1.

Therefore, we additionally examined this possibility in Study 2 (Hypotheses 4a–c), which was partially supported: reputational damage estimation mediated the negative relationship between relational mobility and social media addiction, whereas the motivation to expand the social network mediated the positive relationship. This result supports Hypotheses 2 and 4c.

Additionally, we found a statistically significant main effect of sex in both Studies 1 and 2 in that females were more strongly addicted to social media than males were. Chen et al. [54] demonstrated a difference between sexes in the factors associated with smartphone addiction. They found that females were more likely to be addicted to smartphones as they used social networking services, whereas this relationship was not found for males. Although it is only a speculation, our study demonstrated that females were more strongly addicted to social media, partially because we focused on LINE, a social media especially for connecting with others.

The explorative analysis in Study 2 demonstrated an interesting interaction effect between age and the motivation to expand the social network. Those with a higher motivation to expand the social network were more strongly addicted to social media, and this effect was smaller among older people (*M* + 1*SD*) than younger ones (*M* - 1*SD*). This may be partially because social media usage does not expand the social networks among older people as much as among younger people. According to Kojima (2022) [55], the rate of those who use LINE every day was lower among older people (50s male: 56.3%; 50s female: 69.9%) than among young people (20s male: 76.2%; 20s female: 86.8%). Even when older people try to send messages to their friends through social media, their friends may not use social media. Future research should focus on the differences in the social media environments between various age groups.

In contrast to Hypothesis 4a, loneliness did not mediate a positive relationship between relational mobility and social media addiction. We found no relationship between loneliness and social media addiction (*r* = 0.04, *p* = 0.23). This result is inconsistent with previous studies that have demonstrated a positive relationship between loneliness and social media addiction [23]. This non-significant relationship was surprising in that the COVID-19 pandemic would have strengthened the relationship between loneliness and social media use. Kayis et al. [56] demonstrated that the fear of COVID-19 strengthened loneliness, which in turn strengthened smartphone addiction. Although speculative, the capacity to be alone might have weakened the relationship between loneliness and addiction. As the capacity to be alone is negatively related to social media addiction [57], even when individuals feel lonely, if their capacity to be alone is significant, they will not be strongly addicted to social media. We also found that loneliness was significantly negatively correlated with relational mobility (*r* = -0.26, *p* < 0.01), which is inconsistent with the results obtained by Oishi et al. [50], who found that participants felt lonelier when they were asked to imagine a situation wherein they frequently moved to a different location. Although relational mobility is high in societies in which people move frequently [58], this is not always the case. Even in such societies, some people have fewer opportunities to construct new relationships. This might be the reason that our results, which focused on relational mobility, were inconsistent with those obtained by Oishi et al. [50].

Moreover, in contrast to Hypothesis 4b, when we used SEM techniques and examined the following hypotheses, the model did not fit the data (RMSEA = 0.12). Although the model did not fit the data, the direction of each path was statistically significant and consistent with our hypotheses: (1) people in a lower relational mobility society estimated greater reputational damage (β = -0.21, *p* < 0.01), which strengthened their social media addiction (β = 0.14, *p* < 0.01), whereas (2) people in a higher relational mobility society were more extraverted (β = 0.40, *p* < 0.01), which strengthened their social media addiction (β = 0.12, *p* < 0.01). A reason why our model did not fit the data was the weak relationship between extroversion and social media addiction. Although some studies have demonstrated a positive relationship between extroversion and social media addiction [17], others have indicated no relationship between them [15]. Future studies should examine the factors that moderate the relationship between extroversion and social media addiction.

We also found no relationship between age and addiction in this study. This may be because, compared with studies that have focused on university students [28, 31, 54], the percentage of younger participants in our studies was low. Studies 1 and 2 included 6.84 and 6.20% of individuals in their 20s, respectively, and 18.10 and 17% in their 30s, respectively. Additionally, we did not recruit minors (aged < 18 years). This may be a reason for us not finding a relationship between age and social media addiction.

We also examined the accuracy of reputational damage estimation incurred by ignoring messages, as hypothesized in Hypothesis 3, which was partially supported: people in low relational mobility societies overestimate the reputational damage incurred by ignoring messages. This was consistent with Hypothesis 3. Meanwhile, people in high relational mobility societies also overestimated the reputational damage incurred by ignoring messages, which was inconsistent with Hypothesis 3.

#### 3.3.2. Practical implications

Our results suggest that the antecedent factors of social media addiction differ between high and low relational mobility societies, which implies that interventions for moderating social media addiction differ between high and low relational mobility societies. We demonstrated that people in higher relational mobility societies had a higher motivation to expand social networks, which strengthened their social media addiction. Therefore, moderating this motivation could be effective in preventing social media addiction.

In contrast, we also demonstrated that people in lower relational mobility societies estimated greater reputational damage incurred by ignoring messages, which strengthened their social media addiction. This reputational damage was overestimated. Other relevant studies have found that people who are highly sensitive to rejection are more likely to perceive others’ ambiguous behaviors as intentional rejections [59] and are more strongly addicted to social media [60]. These studies, as well as our results, imply that people overestimate the possibility of being rejected or reputational damage incurred by ignoring messages, which can strengthen their social media addiction. Therefore, correcting this estimation can be effective for lowering social media addiction, especially among those in lower relational mobility societies or those more sensitive to rejection.

One specific intervention can be to provide them with feedback that ignoring messages does not lower their reputation as much as they estimate. For example, by asking students in a class to evaluate those who ignore messages on social media and by showing them the distribution or average of the evaluation score, they would notice that they overestimate the reputational damage. This type of intervention has previously succeeded in changing behavior, such as reducing college students’ alcohol consumption [61]. Students used to excessively consume alcohol based on the incorrect estimation that other students prefer alcohol [62], but by correcting their inaccurate estimation (i.e., by informing them that other students do not prefer alcohol as much as they estimated), their alcohol consumption was decreased [61]. Although these studies were conducted 30 years ago and did not involve social media, we believe that modifying or correcting the estimation of reputational damage can be a novel and important intervention to lower addiction as it focuses on interpersonal miscommunications between social media users, which differs from other interventions that focus on individual aspects (e.g., asking users to reflect on “what social media they used, how long and how they used the social media, their thoughts and emotions related to their social media use” [5]).

#### 3.3.3. Limitations and future work

This study had several limitations. First, it only included participants from Japan. We assumed that even in Japan, different cities have different degrees of relational mobility. Indeed, some studies have surveyed people in Japan and demonstrated the effect of relational mobility on their attitudes [63, 64]. However, Japanese people do not necessarily live in high relational mobility societies because relational mobility in Japan is low [44]. This might be the reason that, inconsistent with the prediction of Hypothesis 3, people from both high and low relational mobility societies overestimated the possibility of earning a bad reputation by ignoring messages. Additional studies must be conducted in higher relational mobility societies, such as the United States, to investigate our hypotheses.

Second, we used a crowdsourcing service to recruit participants from both high and low relational mobility societies. However, it has been demonstrated that some participants recruited via crowdsourcing services answer questions carelessly [65], which might have affected our results. Although we performed an instructional manipulation check and excluded those who did not pass it, additional studies are required to ensure the robustness of our results.

Finally, our study did not sufficiently investigate the effects of COVID-19 on distress or social media addiction. Some studies have focused on the psychological distress caused by the COVID-19 pandemic and demonstrated that it strengthened social media addiction [20, 30]. Therefore, a possible intervention for moderating social media addiction is to lower psychological distress. Tambelli et al. [66] surveyed late adolescents (aged between 18 and 25 years) and demonstrated that those who felt a greater sense of security from their parents or peers exhibited lower COVID-19-related distress. Lowering distress by constructing good relationships with parents or peers could weaken social media addiction, at least among late adolescents.

## 4. Conclusion

This study demonstrated that the antecedents of social media addiction differ between high and low relational mobility societies. In Study 1, we demonstrated that people in low relational mobility societies estimate greater reputational damage, but there was no direct relationship between relational mobility and social media addiction. Therefore, in Study 2, we additionally explored the factors that mediate the positive relationship between relational mobility and social media addiction. The results indicated that (1) people in lower relational mobility societies expect higher reputational damage, which strengthens their social media addiction; and (2) people in high relational mobility societies are more motivated to expand their social networks, which strengthens their social media addiction. In addition, both studies demonstrated that people expect greater reputational damage than the actual damage. These results imply that the mechanism of social media addiction differs depending on the social environment: the estimation of reputational damage strengthens social media addiction in low relational mobility societies, whereas the motivation to expand social networks increases social media addiction in high relational mobility societies. Therefore, correcting this damage overestimation would be an effective strategy to moderate social media addiction, especially in low relational mobility societies, whereas reducing the motivation to expand social networks would be effective especially in high relational mobility societies.
